# Effectiveness of Integrated Trauma System Implementation on Road Traffic Fatalities and Injuries in the North of Iran Using an Interrupted Time Series Analysis

**DOI:** 10.34172/jrhs.2025.175

**Published:** 2024-12-25

**Authors:** Enayatollah Homaie Rad, Farideh Sadeghian, Zahra Ghodsi, Shahrokh Yousefzadeh-Chabok, Mohammad Hosein Ranjbar Hameghavandi, Shahriar Ghashghaee, Zahra Mohtasham-Amiri, Hamid Heidari, Gerard O’Reilly, Seyed Mohammad Ghodsi, Vafa Rahimi-Movaghar

**Affiliations:** ^1^Guilan Road Trauma Research Center, Trauma Institute, Guilan University of Medical Sciences, Rasht, Iran; ^2^Sina Trauma and Surgery Research Center, Tehran University of Medical Sciences, Tehran, Iran; ^3^Center for Health Related Social and Behavioral Sciences Research, Shahroud University of Medical Sciences, Shahroud, Iran; ^4^Social Determinants of Health Research Center, Guilan University of Medical Sciences, Rasht, Iran; ^5^School of Public Health and Preventive Medicine, Monash University, Melbourne, Australia; ^6^Emergency and Trauma Centre, The Alfred, Melbourne, Australia; ^7^National Trauma Research Institute, The Alfred, Melbourne, Australia; ^8^Brain and Spinal Cord Injury Research Center, Neuroscience Institute, Tehran University of Medical Sciences, Tehran, Iran; ^9^Department of Neurosurgery, Shariati Hospital, Tehran University of Medical Sciences, Tehran, Iran; ^10^Universal Scientific Education and Research Network (USERN), Tehran, Iran; ^11^Institute of Biochemistry and Biophysics, University of Tehran, Tehran, Iran; ^12^Visiting Professor, Spine Program, University of Toronto, Toronto, Canada

**Keywords:** Integrated trauma system, Traffic, Interrupted time series analysis, Wounds and injuries, Mortality

## Abstract

**Background:** Integrated trauma systems (ITS) have shown potential in reducing traffic crash-related injuries and mortality, although their structure and impact can vary. This study assessed the effectiveness of ITS in Guilan, Iran.

**Study Design:** A retrospective observational study.

**Methods:** Utilizing a cross-sectional analysis, this descriptive-analytical study was conducted in Northern Iran from 2015 to 2019 to compare road traffic crash (RTC) data in Guilan (with ITS) against Mazandaran (without ITS), considering weather as a confounder. The study involved pre- and post-ITS intervention analyses to evaluate changes in RTC, injury, and mortality rates.

**Results:** Before ITS implementation, Guilan’s daily RTC mean was 38.4 (SD=16.7), which significantly decreased to 30.8 (SD=13.7) after the intervention. Conversely, in the control province of Mazandaran, the daily average number of RTCs increased from 37.29 (SD=14.1) to 42.55 (SD=16.4) post-ITS implementation in Guilan. Furthermore, the mortality rate in Guilan showed a marginal decline from 27.74 (SD=12.6) pre-ITS to 26.60 (SD=11.9) post-ITS, indicating the positive impact of the ITS. In contrast, Mazandaran demonstrated a significant increase in mortality from 32.16 (SD=14.5) to 51.75 (SD=15.7). The statistical analysis confirmed a significant reduction in mortality and injury incidence in Guilan at the time of the intervention, with a marked decrease observed post-intervention (*P*<0.001).

**Conclusion:** The findings revealed that RTC prevention is feasible in Iran, with ITS in Guilan significantly reducing RTCs, injuries, and mortalities, underscoring the importance of ongoing efforts to expand ITS components nationally.

## Background

 Road traffic crashes (RTCs) are among the most frequent causes of injury, mortality, and disability, with significant costs for individuals, families, and countries worldwide.^1,2^ According to the World Health Organization report, 93% of the mortality of road traffic injuries (RTIs) have occurred in low- and middle-income countries.^3^

 Despite a notable decrease in the disability-adjusted life year rate for RTIs over the past thirty years in Iran, the corresponding rate for RTIs in 2019 remained approximately 1.5 times higher than the global average. Moreover, the age-standardized incidence, *years of life lost*, and years lived with disability rates per 100 000 for RTIs in Iran were significantly higher compared to global figures.^4^ Iran is one of the highest-ranked countries for vehicular injury incidence.^5^ A study highlighted that Guilan, Golestan, and Mazandaran provinces in Northeast Iran have higher than average traffic-related fatalities due to increased tourism. The findings showed an age-adjusted fatality rate of 33.4 per 100 000 annually in these regions, with 44.7% involving drivers, averaging 39.5 years old.^6^ Guilan is particularly risky for road traffic collisions due to its unique road design, industrial and urban development, and appealing geographic and climate conditions for tourists.^7^

 The World Health Organization highlights the preventability of RTCs and stresses the need for a holistic road safety strategy involving transport, law enforcement, healthcare, and education to protect roads, vehicles, and users. Recommendations include developing safer infrastructure, improving vehicle safety, and enhancing post-accident care, strict law enforcement, and public education on RTC prevention.^8^ While Iran has these components and offers free access to care for RTC victims, their organization lacks effectiveness.^6^

 The implementation of an integrated road accident system implementation is an approach to decreasing road traffic injuries and mortality in the world.^9^ These systems have been implemented in developed regions, but in less developed countries, there is still a lack in the implementation of integrated road traffic surveillance systems.^10,11^ These integrated systems, which are called trauma systems (TS), contain organized multidisciplinary activities in response to injury. However, the structure of these systems is highly different in different regions, and in many countries, they contain both prevention and response to injury.^10,12,13^

 A systematic review reported that only 9 out of 23 high-income countries have a comprehensive nationwide TS, while such systems are scarce in middle- and low-income countries.^14^ Another review showed that implementing TSs significantly improves survival rates for severely injured patients.^15^ The road trauma surveillance system is vital for injury control. High-income countries use police data, while low- and middle-income countries often lack reliable sources.^16^ Countries with national TSs have six times lower trauma death rates than those without such systems.

 Time series analysis aims to develop a model that identifies and corrects the temporal dynamics in data, enabling accurate future predictions. The literature on interventions for controlling RTC-related injuries and deaths in Iran is scarce.^6^ Accordingly, this study seeks to narrow this research gap by evaluating interventions to decrease RTCs and their negative effects. Specifically, this study assesses the effectiveness of an integrated TS (ITS) in reducing RTC, injuries, and fatalities in Northern Iran through time series analysis.

## Methods

 This retrospective cross-sectional study was performed using a time series model. Guilan and Mazandaran are two northern provinces of Iran on the coast of the Caspian Sea. Guilan province TS intervention has covered 14 709 km^2^ area (whole area of Guilan province) (approximately 2 521 000 population in 2018), and Mazandaran has an area of 23 756 km^2^ (approximately 3 280 000 population in 2018).^17^ Both Guilan and Mazandaran are dense provinces, and their transport systems suffer from a high amount of motor vehicles as well as pedestrians and domestic animals on the roads.^18,19^ In these provinces, towns and villages are highly close to each other and close to the road. The Guilan TS model has two levels of public hospitals in all 15 counties (the first level contains 19 hospitals) and Poorsina Hospital of Rasht (the second level). Emergency patients, after the diagnosis by the emergency medical dispatcher, were directly transferred to the second level. In the first phase, the RTC data were collected for both provinces. After RTC, first of all, the police and emergency medical services (EMS) come to the crash scene. Sometimes other emergency organizations, such as the Red Crescent or Fire Organization, help EMS to transfer injured patients to hospitals. More information about Guilan TS is available at https://ts.gums.ac.ir and Supplementary file 1.

 For this study, data were daily sorted to calculate daily traffic crashes, mortality, and injuries from 1 January 2015 to 30 December 2019 in Guilan and Mazandaran. The study began on 21 March 2017, marking the implementation of the ITS in Guilan, which introduced new interventions in its first upgrade phase.

 Data from this study were collected in correspondence with the police. The police have a data bank that registers all cases of road traffic injuries in Iran. Data from Guilan and Mazandaran provinces were extracted from the data bank. Data existed for each event (i.e., crash) and were collapsed to daily rates for analysis using ITS methods. All RTCs and related deaths and injuries were identified and collected in the databank. Due to the potential changes in RTC incidence due to changes in weather conditions, precipitation data were added as potential confounding variables in the model. The daily precipitation data were collected from the Iranian National Weather Organization.

###  Intervention

 The initial idea for upgrading the TS was formed in 2003 with the establishment of the Road Research Center at Guilan University of Medical Sciences. Preliminary measures were expanded in 2006. The participation of the governor and the director of the Plan and Budget Organization was established, and phase one of the intervention had begun. It was included in the integrated collaboration of the organizations to help injured people and public education on local Radio and TV.

 The main core of phase one of the intervention included:

(α) Integration and coordination across stakeholder organizations.
(β) Formation of a specialized working group, the ITS Monitoring Committee, for traffic crash management, holding monthly meetings to discuss challenges, solutions, and unified prevention plans.
(χ) Creation of a digital trauma case library with traffic crash information from each organization for evidence-based policy-making, including databases on demographics, injury characteristics, care processes, and outcomes, linkable by patient ID.
(δ) Scientific analysis of Guilan’s road data to identify and mitigate high-risk points through collaboration with police and relevant bodies, implementing speed control and other safety measures. The meteorological organization analyzed crash weather patterns, providing regular warnings to the committee.
(ε) Holding scientific training programs based on the needs of the audience, stakeholders, and high-risk groups. Education programs were prepared for Provincial Radio and Television by police, the Road Trauma Research Center, EMS, the Road Transport Organization, and the Health Education Department of Health Chancellor of Guilan University of Medical Sciences. Other educational plans were implemented by Red Crescent and the Deputy of Health in rural and less developed regions. The preparation of educational content was mainly with the Road Trauma Research Center affiliated with Guilan University of Medical Sciences. Pamphlets were also sent to the passengers and drivers.

###  Participating organizations 

 Several stakeholder organizations participated in the TS project, including the Road Trauma Research Center affiliated with the Guilan University of Medical Sciences, the Traffic Police, the Road Police, the Prevention Police, and the Applied Studies Police. The other organizations were EMS, Poorsina Hospital (center of trauma), Red Crescent Organization, Highway Organization, Provincial Government, Oil Company, Fire Organization, Forensic Medicine, Road Transport Organization, TV and Radio Organization, and Meteorological Organization.

###  Statistical analyses 

 Before implementation, each part of the plan was analyzed and compared with the literature, and the role of each organization was identified in the workgroup. Analyses of time series trends were conducted to compare the outcome rates before and after March 21, 2017 for both Guilan and Mazandaran provinces, separately.

 Interrupted time series analysis (ITSA) was used for statistical analysis. Each ITSA pattern consists of main variables with the following general content:

 Formula (1): Y_t_ = β_0 _+ β_1_T_t _+ β_2_X_t _+ β_3_X_t_T_t _+ ϵ

 B_0_ is the constant coefficient of each of the variables under consideration, and T denotes the total time period examined, respectively. In addition, X is a dummy variable of the time of making changes (for this study, before the defined timepoint for the implementation of phase one of the ITS, the value of X is 0, and following the same timepoint, the value of X is 1,). Further, X_T_ is the interaction variable or interaction of the time series and the time-point variable when changes are made. Furthermore, ε is the residual of the model and contains those parts of the model that cannot be determined and evaluated by independent predictors.^18^

 The variable Tt represents the time points in the study, ranging from the start to the end of the observation period. It is not a constant but a sequential variable capturing the progression of time. In the case of the negative and significant variable of X, it can be claimed that the implementation of phase one of the ITS has reduced the number of road crashes. In the case of negative and significant interaction coefficient, it can be indicated that phase one of the implementation of the ITS has had a sustainable effect on the reduction of traffic crashes.

 Therefore, the pattern in the study was as follows:

 Formula (2): Y_t_ = β_0_ + β_1_T_t_ + β_2_X_t_ + β_3_X_t_T_t_ + β_4_ weather_t _+ e_t_

 where ‘weather’ is the average daily rainfall in each province, taken from the systems of the National Meteorological Organization.

 The outcome (response) variable of this study contained the daily number of injuries and mortalities (the police data were gathered, sorted, and aggregated to the daily level) and the number of crashes registered by road traffic police each day.

 Guilan and Mazandaran provinces, neighboring each other, were found to share similar traffic flow, educational levels, wealth, and employment types. However, their weather conditions, particularly precipitation levels—a key factor affecting RTCs—differed, making precipitation a critical confounding variable in the analysis. The intervention’s impact was anticipated to be immediate, without needing lags. The Durbin-Watson test was used to check for autocorrelation, while Chow’s test was employed to compare model coefficients at the end of the analysis. The statistical analysis was performed with STATA software.

## Results

 The mean daily number of mortalities and injuries (totally) in Mazandaran province was 43.1 (18.3), while it was 27.1 (12.2) in Guilan province. The mean daily incidence of RTC was 40.14 (15.6) in Mazandaran and 34.17 (15.6) in Guilan. Moreover, the average number of mortalities and injuries before and after upgrading the TS in Guilan province was 27.74 (12.6) and 26.60 (11.9), respectively, while it was 32.16 (14.5) and 51.75 (15.7) for Mazandaran province, respectively. Furthermore, the daily average number of crashes in Guilan province was 38.40 (16.7) before upgrading the TS in Guilan province, while it decreased to 30.79 (13.8) after the intervention. In Mazandaran province (as the control), the daily average number of crashes was 37.29 (14.1) before upgrading the TSs in Guilan, while it increased to 42.55 (16.4) after the intervention (Table 1).

**Table 1:** Descriptive statistics of daily crashes, mortalities, and injuries before and after trauma system implementation

**Variables**	**Mean**	**SD**	**Min.**	**Max.**
**Total data**				
Guilan				
Mortalities and injuries	27.106	12.227	1	78
Crashes	34.166	15.615	1	112
Mazandaran				
Mortalities and injuries	43.060	18.032	5	109
Crashes	40.140	15.613	6	119
**Before trauma system implementation**				
Guilan				
Mortalities and injuries	27.741	12.565	4	78
Crashes	38.403	16.719	4	112
Mazandaran				
Mortalities and injuries	32.163	14.4575	5	97
Crashes	37.290	14.109	7	84
**Just after trauma system implementation**				
Guilan				
Mortalities and crashes	26.600	11.933	1	75
Crashes	30.788	13.778	1	80
Mazandaran				
Mortalities and injuries	51.747	15.740	21	109
Crashes	42.554	16.358	11	119

*Note*. SD: Standard deviation; Max: Maximum; Min: Minimum.

###  Results of Guilan as a case

**Figure 1:**
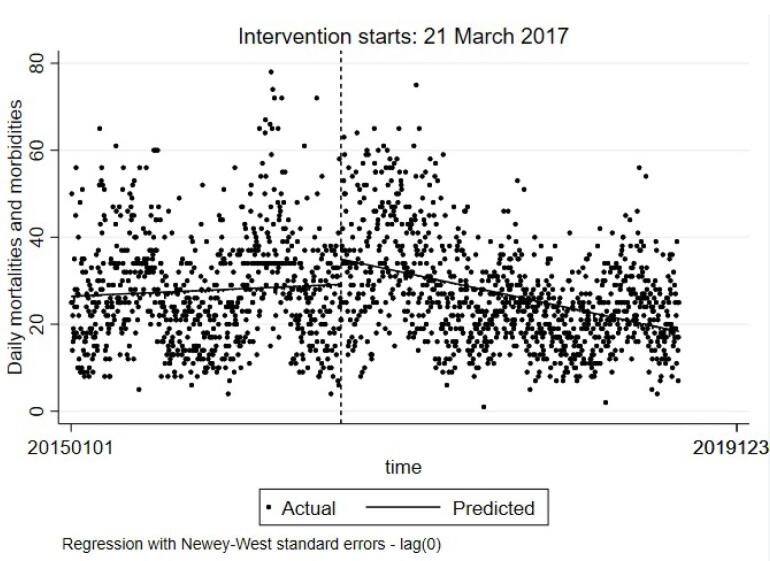
ITSA model for mortalities and injuries due to road traffic crashes in Guilan province (a comparison between January 23, 2015 and December 30, 2019). *Note*. ITSA: Interrupted time series analysis

Table 2 presents the interrupted time series model to investigate the impact of the ITS intervention on mortality and injuries in Guilan province. Based on the results, β1 (for T) did not have a significant relationship with mortality and injury (*P*= 0.078), while β2 (for X) had a positive relationship with mortality and injury, indicating that the incidence of mortality and injury increased in the post-intervention phase compared to the pre-intervention phase (coefficient = 5.8, *P* < 0.001). However, β3 (for XT) was negative and significant (coefficient = -0.019, *P* < 0.001); this may imply that the upgrade of the TS in Guilan province had a sustainable effect on both mortality and injury rates since RTC precipitation was adjusted in the model but it had no significant relationship with mortalities and injuries. Figure 1 displays the graphic form of the ITSA model. As shown, the predicted line of mortalities and injuries had a low ascending slope, while after the intervention, the predicted line was descending.

**Table 2:** Interrupted time series model to show the effects of the upgrading trauma system on mortalities and injuries in Guilan province

**Variables**	**Coefficient**	**SE**	**t**	***P *** **value**	**Lower limit**	**Upper limit**
Weather^*^ (β4)	-0.01	0.019	-0.73	0.465	-0.05	0.02
β1	0.00	0.00	1.76	0.078	-0.00	0.00
Β2	5.80	1.22	4.74	0.000	3.40	8.21
Β3	-0.01	0.00	-8.66	0.000	-0.02	-0.01
Constant	26.45	0.85	30.86	0.000	24.77	28.13

*Note*. SE: Standard error. ^*^This is the adjusted variable of precipitation.

Table 3 provides the ITSA model for the daily number of crashes in Guilan province. According to the findings, β1 did not have any significant relationship with the number of crashes; β2 was positive and significant, representing that the number of crashes was increased at the time of upgrading TS in Guilan province. Β3 had a negative and significant relationship with the number of crashes, confirming a sustainable effect of upgrading the TS plan on decreasing the number of RTCs. The Durbin-Watson test results were 2.100 and 2.148 for the first and second models, respectively; in other words, the estimated models did not have autocorrelation.

**Table 3:** The ITSA model for number of crashes in Guilan province

**Crashes**	**Coefficient**	**SE**	**t**	***P *** **value**	**Lower limit**	**Upper limit**
Weather^*^ (β4)	0.02	0.02	0.86	0.391	-0.02	0.07
β1	-0.00	0.00	-1.22	0.224	-0.00	0.00
Β2	4.87	1.41	3.44	0.001	2.09	7.65
Β3	-0.01	0.00	-6.73	0.000	-0.02	-0.01
Constant	39.52	1.24	31.7	0.000	37.08	41.97

*Note*. SE: Standard error; ITSA: Interrupted time series analysis. *This is the adjusted variable of precipitation.

**Figure 2:**
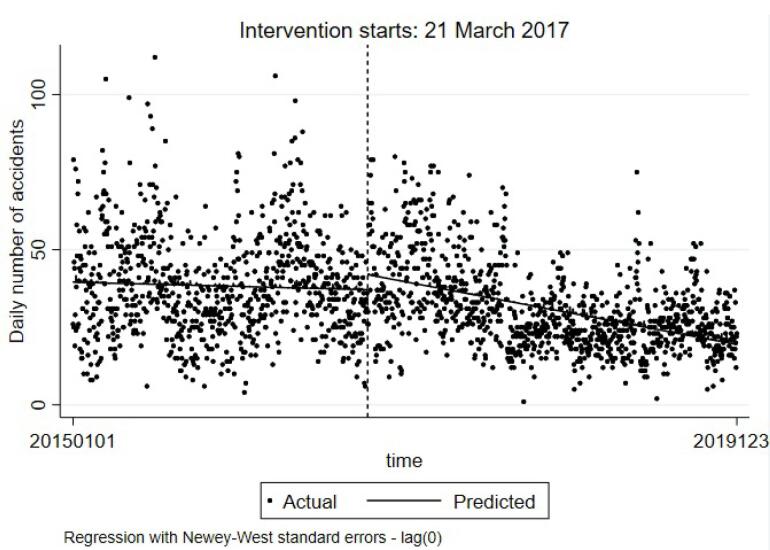
ITSA Model for number of traffic crashes in Guilan province. *Note*. ITSA: Interrupted time series analysis

Figure 2 illustrates the ITSA model for the number of crashes. The predicted line of crashes in the pre-intervention phase had a low descending slope, while after the intervention, the predicted line was descending with a slope of greater magnitude.

###  Results of the study in Mazandaran as a control 

Table 4 lists the number of mortalities and injuries as well as the number of RTCs in Mazandaran province if it is hypothesized that the intervention was performed in Mazandaran similar to Guilan province. Based on the obtained data, the β1 was positive and significant for mortality and injury incidence due to RTCs in Mazandaran province (coefficient = 0.007, *P* = 0.001), while it was negative for RTCs (coefficient = -0.0275, *P* < 0.001). The β2 was positive and significant in both models. The β3 was -0.012 (*P* < 0.001) for the mortality and injury model, indicating that at the time of implementing the TS program in Guilan, a decrease occurred in mortalities and injuries due to RTCs in Mazandaran province. However, the coefficient of XT (*β*3) was smaller in Mazandaran province compared to Guilan. The *β*3 was 0.039 for the crash model in Mazandaran province and it was significant (*P* < 0.001), highlighting that the number of crashes has increased in Mazandaran at the time of implementation of the TS plan in Guilan province. The Durbin-Watson test was 2.085 and 2.172 for the first and second models, respectively, in Mazandaran.

**Table 4:** Number of mortalities and injuries as well as road traffic crashes in Mazandaran province as the control group

**Variable**	**Mortalities and injuries**			**Crashes**		
	**Coefficient**	**SE**	***P *** **value**	**Coefficient**	**SE**	***P *** **value**
Weather^*^ (β4)	-0.09	0.06	0.14	-0.05	0.0632	0.377
β1	0.00	0.00	0.00	-0.02	0.0020	0.000
Β2	19.70	1.52	0.00	10.52	1.1857	0.000
Β3	-0.01	0.00	0.00	0.03	0.0025	0.000
Constant	29.37	0.94	0.00	48.46	1.0667	0.000

*Note*. SE: Standard error. ^*^This is the adjusted variable of precipitation.

###  Comparison of the results of two provinces

Table 5 summarizes the results derived from comparing the coefficients of the two models. Based on the results, the *βs* of the model were compared to each other using the Chow test. The hypothesis of having similar *βs* was rejected for each of the *β1*,* β2*,and* β3*, in turn. Thus, the slopes, changes in slopes after the time of intervention, and the interaction between time and slopes differed between Guilan and Mazandaran provinces.

**Table 5:** Comparing Guilan and Mazandaran regression model coefficients using Chow test

**Model/Hypothesis**	**Chow test χ2**	***P*** ** value**
Mortalities and injuries		
β1 _case _= β1 _control_	4.28	0.038
Β2 _case _= β2 _control_	149.96	0.000
Β3 _case _= β3 _control_	9.64	0.001
Crashes		
β1 _case _= β1 _control_	59.02	0.000
Β2 _case _= β2 _control_	8.03	0.004
Β3 _case _= β3 _control_	222.34	0.000

**Figure 3:**
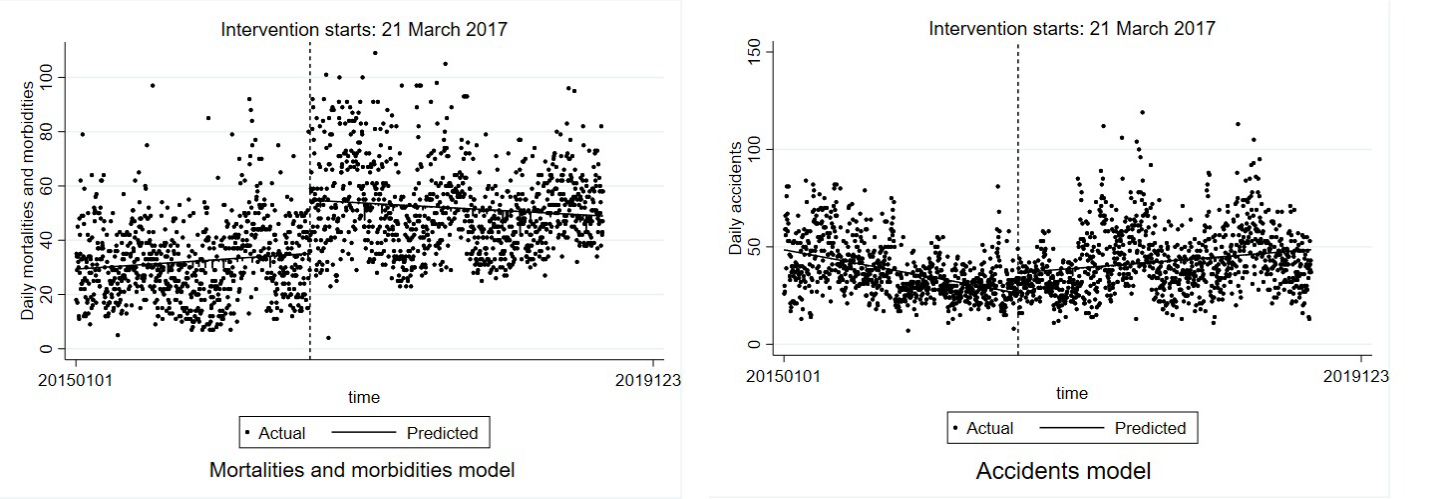
ITSA models for mortalities and injuries due to road traffic crashes (**A**) and crashes (**B**) in Mazandaran province. *Note*. ITSA: Interrupted time series analysis

Figure 3 depicts the ITSA models for mortalities and injuries (A) and crashes (B) in Mazandaran province. As shown, after the intervention, the slope of the mortality and injury model did not change highly, and the slope of the predicted line was not as steep as the Guilan model; however, the Chow test showed that the slopes were different. For the accident model, the predicted line was ascending after the time of implementing phase one of upgrading TS.

## Discussion

 Our study evaluated the ITS effectiveness in North Iran (2015-2019), focusing on stakeholder coordination, crash registration, and educational programs, using time series analysis. The results indicated a significant reduction in RTCs, injuries, and deaths with ITS implementation. In Guilan, average RTCs dropped from 38.40 to 30.79, and mortality/injury rates decreased from 27.74 to 26.60. Conversely, Mazandaran, as a control, experienced increases at the intervention time. Despite minimal changes in the ITS model, the impact was significant, expected due to unchanged factors such as road quality, vehicle safety, and population density.^20,21^

 A systematic review study reported the establishment of eight of the nine TSs or some component of the improved outcomes to 15%–20% in the survival rate among seriously injured patients^15^. Another study in Iran indicated that educational planning interventions can be influenced by a decrease in road traffic injuries.^8^

 The findings of Cales et al demonstrated that a TS in Los Angeles reduced death rates from 34% to 15%, with a significant difference in deaths between non-trauma (54%) and trauma (4%) centers, and the median age of RTC fatalities increased from 22 to 27 years.^22^ Our study also reported a reduction in RTCs by 19.82%. Educational programs globally, including in England, North America, and India, have decreased RTCs by up to 17.9% and traffic violations, suggesting that such interventions can produce safer drivers and roads.^23-26^ However, data on traffic crash prevention in developing countries are scarce, with a Hungarian study showing a 13% decrease in daytime traffic crashes with mandatory daytime lights.^6,27^

 Post-intervention, mortality and injury rates in Guilan decreased by 4.1% from 27.74 to 26.60, while Mazandaran and other Iranian provinces experienced increases in traffic accident injuries. Studies highlight the effectiveness of safety interventions globally. In Indonesia, helmet usage reduced head injuries from 52% to 32%,^28^ and in Taiwan, helmet laws led to a 6,240 increase in quality-adjusted life-years due to fewer motorcycle crash head injuries.^29^ Johannesburg observed a significant drop in traffic crash injuries from 48 to 20 after enforcing speed limits.^30^ Moreover, Singapore reported a 52% and 66% reduction in severe and trivial injuries, respectively, over nine years due to school road safety education programs.^31^ Additionally, some studies supported the effectiveness of speed cameras and management in reducing RTC mortality and injury.^32,33^ Khorasani-Zavareh et al identified human factors, transportation systems, and organizational coordination as barriers in Iran, advocating for an integrated RTI prevention system and a shift in stakeholders’ prevention attitudes.^34^ A systematic review recommended a comprehensive set of interventions for traffic crash prevention.^35^

 Our intervention included a set of integration and coordination of RTC stakeholder organizations, as well as improved registration and educational programs for updating the TS. To the best of our knowledge, it was the first intervention in Iran. One strength of this study is that, according to our results the prevention of RTC is reachable in developing countries. The establishment of an ITS was successful but the most important issue was the durability of this system.

 However, our study had some limitations. This study was a cross-sectional survey. In addition, police reports were used as a source of RTC, death, and injury registration; thus, there were no data on mortality and injury separately or data on death on the way to the hospital or in the hospital. Furthermore, it was assumed that the type and quality of roads in the two provinces did not differ significantly over the 5 years. Moreover, the amount of traffic was assumed to be relatively constant between the provinces during the study period. However, changes in these variables can be significant, such as traffic patterns increasing proportionally on Fridays and public holidays in both provinces. Additionally, the only climatic variable considered in this study was the amount of precipitation, which typically differed between the two provinces. Other weather-related factors, such as temperature, wind speed, and visibility, were not included in the analysis.

 Future research should examine Guilan province’s success to identify factors influencing RTC outcomes in similar Northern Iranian provinces such as Mazandaran and Golestan. It is recommended that future studies investigate data discrepancies and the feasibility of replicating Guilan’s intersectoral cooperation and ITS in other regions.

**Highlights**

- The integrated trauma system (ITS) improved road safety and reduced traffic injuries and deaths.
- The ITS in Guilan province significantly reduced daily road traffic crashes (RTCs).
- RTC prevention is achievable in developing countries.

## Conclusion

 The implementation of the ITS in Guilan province led to a significant reduction in daily RTCs, injuries, and fatalities. The intervention demonstrated a clear positive impact, contrasting with the control province of Mazandaran, where both RTCs and mortality rates represented an increase. The statistical analysis confirmed the effectiveness of ITS in improving road safety and reducing the incidence of traffic-related injuries and deaths. These findings support the potential benefits of expanding ITS to other regions to enhance public health outcomes and road safety.

## Competing Interests

 Potential conflict of interests may exist for authors affiliated with the Guilan Road Trauma Research Center involved in the Guilan TS project. Nonetheless, the data sourced from the traffic police database are conflict-free. All analysis and data collection phases were supervised by authors without any conflict of interests.

## Ethical Approval

 The Ethics Committee of Tehran University of Medical Sciences approved the study (reference number IR.TUMS.VCR.REC.1398.798).

## Funding

 This work was financially supported by Tehran University of Medical Sciences (grant number is 98-02-38-41757).

## Supplementary Files

Supplementary file 1. Guilan trauma system phases and collaborating organizations.
