# Supplementary material for: Effectiveness of Integrated Trauma System Implementation on Road Traffic Fatalities and Injuries in the North of Iran Using an Interrupted Time Series Analysis
Source: J Res Health Sci. 2024 Dec 25;25(1):e00640. doi: 10.34172/jrhs.2025.175 (PMC11833496; doi:10.34172/jrhs.2025.175)
Supplement: Supplementary file 1 — Guilan trauma system phases and collaborating organizations. [file jrhs-25-e00640-s001.pdf]

JRHS
Journal of Research in Health Sciences
doi:10.34172/jrhs.2025.175
JRHS 2025; 25(1):e00640

Supplementary file 1. Guilan Trauma System Phases and Collaborating Organizations

Implementation	Collaborating Organizations
**Prevention**	
Analyzing accident data and determining high-risk points and patterns of accidents based on seasons and occasions (Nowruz and the like) for the establishment of monitoring and relief devices	Guilan Road Trauma Research Center, Road Police, Transport Police, EMS, Red Cresset, Firefighter, and Meteorological Organization
Compiling, designing, and printing integrated and standard educational content to promote the traffic culture of the community in the form of books as well as cyberspace channels	Guilan Road Trauma Research Center
Holding special workshops and conferences for motorcyclists in the province in the field of helmet use with incentives and punishment	Guilan Road Trauma Research Center, Road Police, Transport Police, and Transport Organization
**Pre-hospital**	
Holding PHTLS course for emergency personnel 115	EMS
Determining on-sense deployment and relief protocols and compiling and producing standard model training videos in the event of accidents	Guilan Road Trauma Research Center, Road Police, Transport Police, EMS, Red Cresset, Firefighter, and Meteorological Organization
Conducting BLS-related training for part of the traffic police agents	Guilan Road Trauma Research Center, Road Police, Transport Police, and EMS
Establishing 115 emergency software systems and provincial dispatch along with equipping all ambulances with GPS	Guilan Road Trauma Research Center, and EMS
**Hospital**	
Establishing a committee to improve the quality of trauma care services in the University Vice-Chancellor for Treatment, as well as mortality committees for accident trauma patients in hospitals	Guilan Road Trauma Research Center, Deputy of Treatment, Poorsina Hospital, and General hospitals
**Infrastructure**	

Establishing a hospital death registration system for accidental patients to diagnose preventable deaths	Guilan Road Trauma Research Center, Poorsina Hospital, and General hospitals
Holding triage courses and dealing with trauma patients for doctors and nurses of hospital emergencies	Guilan Road Trauma Research Center, and Deputy of treatment
Establishing a specialized working group for the prevention and management of traffic accidents and holding regular and continuous monthly meetings to monitor the situation of accidents and responsible agencies in the province, as well as creating integration in the implementation of measures with the presence of 31 related agencies	Guilan Road Trauma Research Center, and 31 provincial organizations
**Infrastructure**	
Establishing a comprehensive system of trauma registration system in the four phases of the trauma system and registration of information of the responsible devices	Guilan Road Trauma Research Center
Designing and compiling specific metrics for provincial devices to monitor performance and predict disaster points	Guilan Road Trauma Research Center

Note. PHTLS: Pre-hospital trauma life support; BLS: Basic life support.
